# Semi-supervised Adversarial Domain Adaptation for Seagrass Detection Using Multispectral Images in Coastal Areas

**DOI:** 10.1007/s41019-020-00126-0

**Published:** 2020-06-02

**Authors:** Kazi Aminul Islam, Victoria Hill, Blake Schaeffer, Richard Zimmerman, Jiang Li

**Affiliations:** 1 Department of Electrical and Computer Engineering, Old Dominion University, Norfolk, VA USA; 2 Department of Ocean, Earth and Atmospheric Sciences, Old Dominion University, Norfolk, VA USA; 3 Office of Research and Development, U.S. Environmental Protection Agency, Durham, NC USA

**Keywords:** Deep convolutional neural network, Seagrass detection, Domain adaptation

## Abstract

Seagrass form the basis for critically important marine ecosystems. Previously, we implemented a deep convolutional neural network (CNN) model to detect seagrass in multispectral satellite images of three coastal habitats in northern Florida. However, a deep CNN model trained at one location usually does not generalize to other locations due to data distribution shifts. In this paper, we developed a semi-supervised domain adaptation method to generalize a trained deep CNN model to other locations for seagrass detection. First, we utilized a generative adversarial network loss to align marginal data distribution between source domain and target domain using unlabeled data from both data domains. Second, we used a few labelled samples from the target domain to align class specific data distributions between the two domains, based on the contrastive semantic alignment loss. We achieved the best results in 28 out of 36 scenarios as compared to other state-of-the-art domain adaptation methods.

## Introduction

Seagrasses create critically important marine ecosystems that provide food to marine animals and humans, stabilize the sea bottom, and absorb carbon dioxide from the environment. Seagrass can be found in coastal areas all over the world [1]. Previous assessments of seagrass distributions from remotely sensed imagery have mostly been performed manually by domain experts [2], although various automated classification methods are now being explored [3]. Our previous work showed that deep convolutional neural network (CNN) models can effectively detect seagrass in multispectral images if the models were trained with enough labelled data [4, 5].

Deep CNN models usually require a large number of labelled training data to achieve competitive results. For seagrass quantification, these labelled data are obtained by in situ observations that are time consuming and labor intensive. Consequently, it can be difficult to collect enough labeled data to train a separate model for each location. However, a well-trained deep CNN model at one location may fail at another location if seagrass density distribution shifts from source domain to target domain. This happens due to the change of appearance/distribution of seagrass from one location to another. Our previous models degraded if directly applied to different locations for seagrass detection [4, 5].

For seagrass detection, we usually have a large amount of unlabeled data for a given new location and it is possible to obtain limited labeled data by domain experts. In this study, we propose a novel domain adaptation approach that uses both unlabeled data and a few labeled samples to learn an effective classifier for new locations. First, we utilized an unsupervised adversarial domain adaptation approach to adapt target domain representation to mimic source domain representation so that the classifier trained in source domain may work in target domain. In the unsupervised domain adaption step, we do not use any labeled samples from the target domain to solve the domain adaptation problem. Second, we utilized a supervised approach with the contrastive semantic alignment loss to learn domain invariant representations between source and target domains. The first step aligns marginal distribution between domains and the second step aligns class specific distributions using a few labeled samples from target domain. The contrastive semantic alignment loss consists of semantic alignment and separation losses. Here, the semantic alignment loss keeps the same class samples from different domains as close as possible. The class separation loss tries to put different class samples from different domains as far as possible. The proposed domain adaptation approach optimizes target domain embedding function to create a simple classifier that can work effectively in the target domain.

**Fig. 1:**
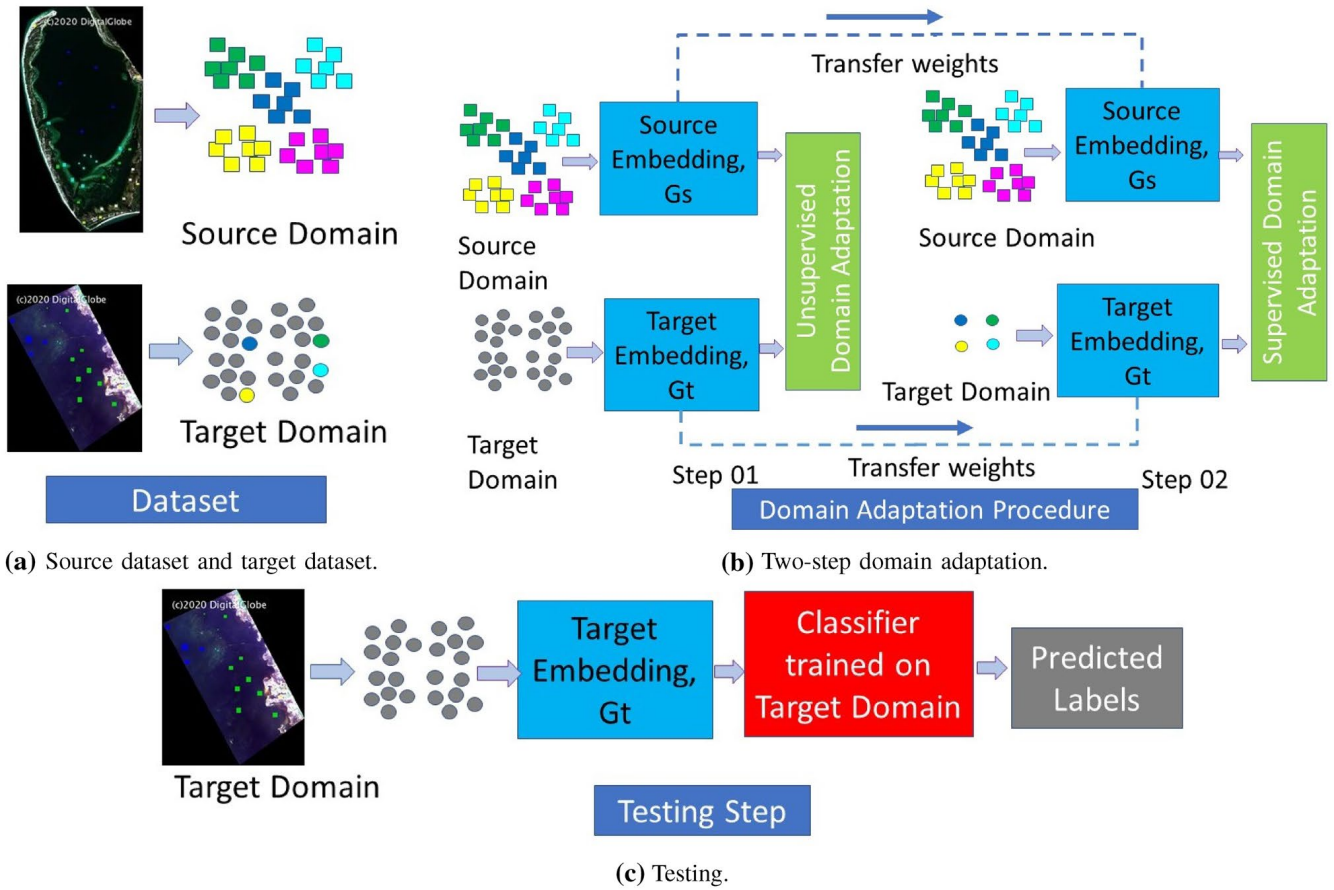
Diagram of the proposed domain adaptation model for seagrass detection. **a** Datasets from both domains where colored samples are labelled, while gray samples are unlabelled. **b** Unsupervised adversarial adaptation and supervised contrastive semantic alignment between target and source domains. **c** The adapted model used for seagrass detection in target domain

Contributions of our proposed approach are:

- A novel approach that uses both unlabeled and a few labeled samples in the target domain to learn a domain invariant embedding for domain adaptation. It can utilize a large amount of unlabeled data for efficient training.
- To the best of our knowledge, this is the first attempt and successful system that can generalize deep CNN models for seagrass detection from one location to another.

The paper is structured as follows: Section 2 discusses the relevant literature. Sections 3 and 4 describe the proposed method and experimental setup. Sections 5 and 6 present results and discussions, respectively, and Sect. 7 summarizes conclusions.

## Related Work

### Seagrass Distributions Mapping

Automated systems to map seagrass distribution in multispectral satellite images have been developed. Traganos et al. proposed a support vector machine (SVM) approach to map the Mediterranean seagrass distribution in Greece utilizing Sentinel-2 satellite imagery [6, 7]. Lions et al. utilized field survey data and multi-spectral image data from the QuickBird satellite for seagrass mapping in shallow coastal water [8]. Different data sources including Landsat [9], IKONOS [10–12], Quickbird [13] and WorldView-2 satellite image sensors [4, 5, 14–16], and different machine learning models such as decision trees, naive Bayes, SVMs [9], maximum likelihood [10, 11, 15, 16] and deep capsule network [4, 5] have been utilized for effective seagrass distribution mapping. However, no model can be directly applied to new locations successfully without adaptation.

### Deep Learning

Deep learning models are a subset of machine learning methods which were inspired to mimic mammal’s vision system. A typical deep learning model consists of multiple layers of feature extraction processing units named as “neurons”. During training, these neurons learn to extract useful features from data to perform classification or regression. Deep learning has been successfully applied in image classification [17, 18], image segmentation [19], image super-resolution [20–22], hyperspectral images[23], object detection [24], speech recognition [25], audio classification [26], computer-aided medical diagnosis [27, 28], medical imaging [29, 30] and cybersecurity [31–33]. Among different deep learning models, deep CNN is the most popular model and more details are provided in a comprehensive survey by Alom et al [34]. A deep CNN model scans input image using a set of trained filters to search for matched patterns contained in the filters. Each layer in the deep CNN model contains a number of trained filters. A layer close to input searches for simple patterns such as edges with different orientations and layers adjacent to output try to match more class-specific patterns to conduct classification. This hierarchy feature extraction mechanism is key to the success of CNN. Popular deep vision CNN models include AlexNet [17], VGG-net [35], Resnet [18], Dense-net [36] and inceptionV3 [37]. Deep CNN has also been applied for seagrass detection in our previous studies [4, 5]. Deep learning models include feature extraction in the optimization loop and achieve state-of-the-art performances in many applications [34]. However, one challenge of deep learning models is they require large training data to achieve competitive performances, making adaptation of deep learning models between domains difficult.

### Domain Adaptation

Domain adaptation techniques can be applied if there are not enough labeled data available to train a deep learning model from scratch in a new domain. In domain adaptation, a model in source domain is first trained using available large training dataset. A domain adaptation method is then applied to adapt the trained model to a new domain (named as target domain) w/o a few labeled samples from the target domain. Tzeng *et. al* proposed an unsupervised domain adaptation method that used the adversarial loss to match source and target domain distributions [38]. Motiian *et. al* proposed a semi-supervised approach for domain adaptation which used the Siamese architecture for domain adaptation [39]. This model learned an embedding function for source and target data where the two domains were semantically aligned and different classes were maximally separated.

**Fig. 2:**
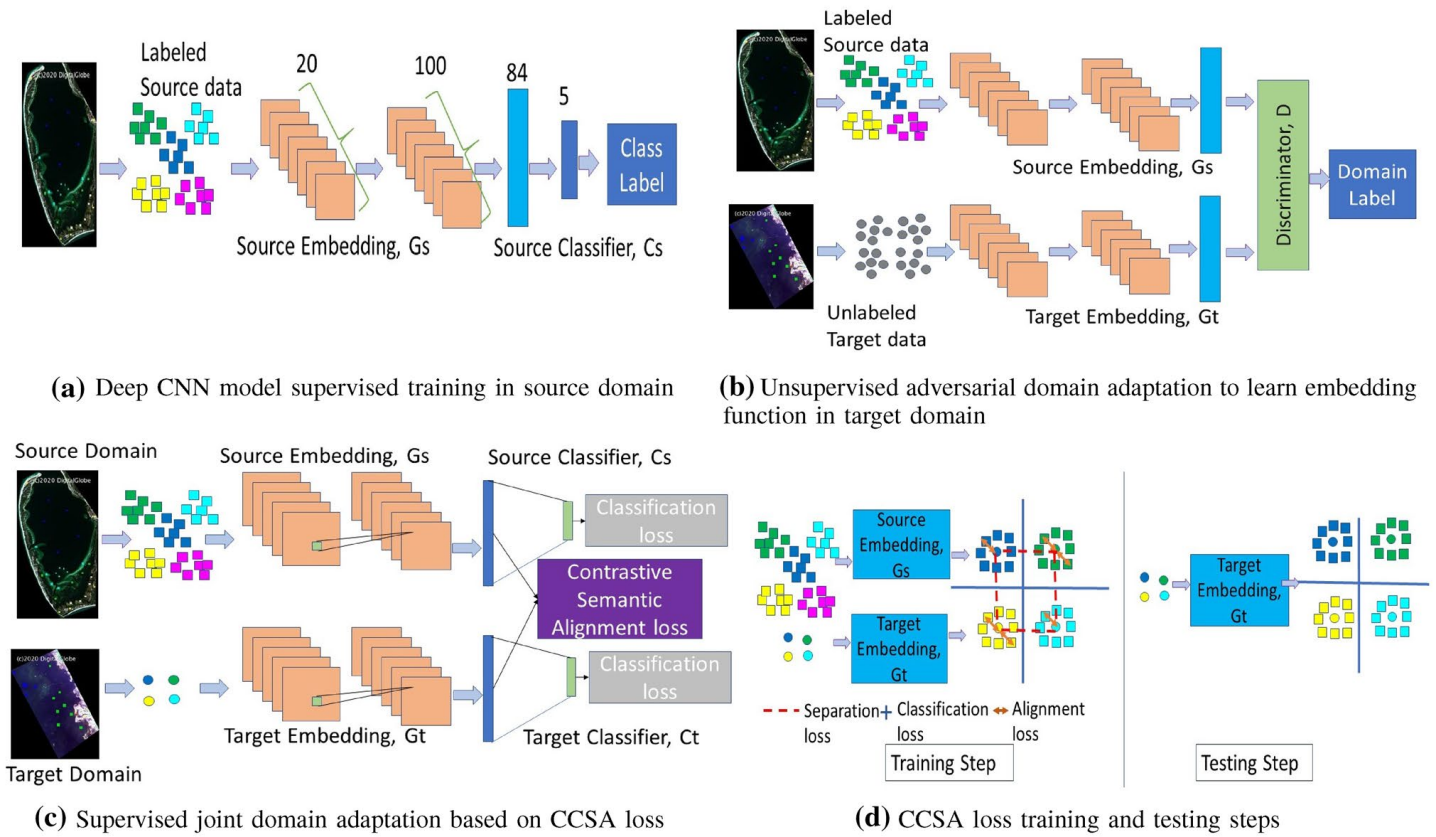
Proposed semi-supervised domain adaption procedure

## Proposed Model

### System Diagram

The diagram of the proposed domain adaptation method for seagrass detection is shown in Fig. 1. There are enough labelled data in the source domain to train a deep CNN model for seagrass detection, while only a few labelled samples in target domain as shown in Fig. 1a. The trained CNN model contains multiple convolutional layers for feature extraction and a fully connected layer for classification. These convolutional layers essentially learn an embedding function, and the fully connected layer takes its outputs for classification. Our proposed system uses two steps to adapt the embedding function trained in source domain to target domain as shown in Fig. 1b. In the first step, the proposed model uses unlabeled samples from both domains to modify the target embedding function while keeping the source embedding fixed, so that the outputs from both embedding functions have a similar distribution. In the second step, the proposed model pair labelled samples from source domain and a few labelled samples from target domain were used to align class specific distributions among both domains. Once the target embedding function is adapted, a simple classifier can be trained using the few labelled samples from the target domain to perform seagrass detection on the remaining target domain (Fig. 1c).

### Model Architecture

Figure 2 shows the domain adaptation procedures of the proposed method. We first train a deep CNN model in the source domain with labeled data (Fig. 2a), where the CNN model learns an embedding function, $G_s$, named as source embedding function, and a simple classifier, $C_s$, for seagrass detection. In the target domain (Fig. 2b), we first use unlabeled data samples from both domains to adapt the target embedding function with a genarative adversarial network (GAN) loss such that the discriminator cannot tell which domain an embedding comes from. This step will align marginal data distributions $p(G_s(x^s))$ and $p(G_t(x^t))$ of the source and target domains. In Fig. 2c, we utilize a few labeled samples from target domain with a classification and a contrastive semantic alignment loss to further adapt the target embedding function such that the class specific data distributions $p(G_s(x^s)|y)$ and $p(G_t(x^t)|y)$ from the two domains are aligned after embedding. Figure 2d illustrates the training and testing steps for class specific alignment. We will detail each of the steps in the following subsections.

### Deep CNN Model Training in Source Domain

Let $D_{s}=\{X^{s}, Y^{s}\}$ and $D_{t}=\{X^{t},Y^{t}\}$ denote source and target domain datasets, and we assume that there are limited amount of labeled samples available in target domain. A source domain deep CNN model is trained with the following classification loss (Fig. 2a),

1 $$\begin{aligned} L_c(f_s)=E[l(f_s(X^s),Y^s)] \end{aligned}$$

where $f_s$ is a classifier to be trained, *E* denotes the expectation function, and *l* denotes any related loss functions.

A classifier, *f*, can be modeled as two functions as $f=G \circ C$, where *G* is the embedding function from the input image *X* to embedding space and *C* is the function for predicting the class label from the embedding space. So $f_s=G_s \circ C_s$ and $f_t=G_t \circ C_t$ denote the deep CNN model in source domain and target domain, respectively.

### Adversarial Discriminative Domain Adaptation

By following the idea in Tzeng et al. [38], we utilize the GAN loss to adapt the embedding function $G_s$ in source domain to target domain. It is assumed that we have source image ${X}^s$ with label ${Y}^s$ from source domain distribution $p_{s}(x, y)$, and image ${X}^t$ from target domain where we do not have any label information. This unsupervised domain adaptation step tries to learn a target embedding function $G_t$ based on $G_s$ and unlabeled data from both domains. $G_t$ and *D* in Fig. 2b are trained by MinMax optimization with the GAN loss $L_{\rm{adv}_D}(X^{s},X^{t},G_{s},G_{t})$,

2 $$\begin{aligned} L_{\rm{adv}_D}\left( X^{s},X^{t},G_{s},G_{t}\right) &=E_{x^{s} \sim X^{s}}\left[ {\rm{log}}D(G_{s}(x^{s}))\right] \nonumber \\&-E_{x^{t}\sim X^{t}}\left[ {\rm{log}}\left( 1-D\left( G_{t}(x^{t})\right) \right) \right] \end{aligned}$$

where *D* is the discriminator used in the GAN model [40] and works as a classifier trained by the cross-entropy loss. The source domain samples are labeled as ‘1’ and target domain samples labeled as ‘0’. The discriminator, *D*, distinguishes whether a sample belongs to source domain or target domain. The target embedding function $G_t$ modifies its parameters using following generator loss,

3 $$\begin{aligned} {\rm{Min}}_{G_{t}} L_{\rm{adv}_G}\left( X^{s},X^{t},D\right) = -E_{x^{t} \sim X^{t}}\left[ {\rm{log}}D(G_{t}(x^{t}))\right] \end{aligned}$$

This is similar to the standard GAN loss where $G_t$ modifies its weights to mimic source domain sample embeddings to fool the discriminator, *D*. During training, we keep $G_s$ fixed while changing $G_t$.

### Classification and Contrastive Semantic Alignment

If there is a distribution shift between source and target domains, the source deep CNN model will not perform well in the target domain. We utilize a few labeled samples in the target domain and some labeled samples in the source domain to jointly adapt $G_s$ and $G_t$ using the classification loss and the contrastive semantic alignment (CCSA) loss proposed by Motiian et al. [39] as shown in Fig. 2c),

#### Classification Loss

We define the classification loss as

4 $$\begin{aligned} L_C(G \circ C)=E[l(f(X),Y)] \end{aligned}$$

This loss function is minimized in the source domain and target domain, respectively, with the selected labeled samples from corresponding domain. This step will separate samples from different classes in both source and target domains, respectively.

#### Contrastive Semantic Alignment (CSA) Loss

To align class specific embedding between source and target domains, we use the CSA loss to jointly adapt $G_t$ and $G_s$. The CSA loss in target domain contains two components and can be described as

5 $$\begin{aligned} L_{\rm{CSA}}(G_t)=L_{\rm{SA}}(G_t)+L_{\rm{CS}}(G_t) \end{aligned}$$

where $L_{\rm{SA}}(G_t)$ is the semantic alignment loss and $L_{\rm{CS}}(G_t)$ is a class separation loss. $L_{\rm{SA}}(G_t)$ is computed as,

6 $$\begin{aligned} L_{\rm{SA}}(G_t)=\sum \limits _{a=1}^{N_c}{d(p(G_s({X}_a^s)),p(G_t({X}_a^t)))} \end{aligned}$$

where $N_c$ is the number of class label, $X_a^s=X^{s}/\{Y=a\}$ and $X_a^t=X^t/\{Y=a\}$ are conditional random variables. *d* is a distance metric between the distribution of $X_a^s$ and $X_a^t$. This semantic alignment loss tries to map source domain and target domain data samples as close as possible if they carry the same class label. However, there is no guarantee that samples from different domains with different labels will be mapped as far as possible in the embedding space. To overcome this challenge, the class separation loss $L_{\rm{CS}}(G_t)$ is computed as

7 $$\begin{aligned} L_{\rm{CS}}(G_t)=\sum \limits _{a,b|a \ne b}{k(p(G_s({X}_a^s)),p(G_t({X}_b^t)))} \end{aligned}$$

where *k* is a similarity matrix which adds a penalty when the distribution of $X_a^s$ and $X_b^t$ are close to each other. This encourages samples with different labels from different domains to be mapped as far as possible in the embedding space. Figure 2d shows the working mechanism of the CSA loss.

During training, the semantic alignment loss (orange arrows) keeps the same class samples from different domains as close as possible. The class separation loss (red dashed line) tries to put different class samples from different domains as far as possible. The classification loss (blue solid line) ensures high classification accuracy in the embedding space. During testing, we use the trained target mapping function to put the unseen target samples into domain invariant space. The overall classification and contrastive semantic alignment loss becomes

8 $$\begin{aligned} L_{\rm{CCSA}}(G_t)=L_{C}(G_t \circ C_t)+L_{\rm{SA}}(G_t)+L_{\rm{CS}}(G_t) \end{aligned}$$

Equations (5)–(8) are used to optimize $G_t$. A similar set of equations are used to optimize $G_s$ such that both embedding functions are jointly adapted.

We paired each labeled sample in target domain with randomly selected labeled and unlabeled samples in source domain to compute the loss in Eq. (8), where *d*(, ) in Eq. (6) is Euclidean distance in the embedded space and *k*(, ) in Eq. (7) is a similarity measure defined between samples.

### Loss Function Computation

The semantic alignment loss and class separation loss are defined as distance or similarity between distributions. It is not easy to estimate conditional distribution for each class given just a few labelled samples in target domain. Following the method described in [39], we compute the semantic alignment loss as

9 $$\begin{aligned} {d(p(G_s({X}_a^s)),p(G_t({X}_a^t)))}= \sum \limits _{i,j}{d(G_s(x_i^s),G_t(x_j^t)))} \end{aligned}$$

where $(x_i^s, x_j^t)$ are all paired labelled samples in source and target domains. Each labelled sample in target domain is paired with many selected labelled samples of the same class in source domain such that $y_j^t=y_i^s=a$. It helps a single labeled target sample to be paired with many source labelled samples and force target labelled samples to be mapped as close as possible to the same class samples in source domain. The class separation loss is calculated as

10 $$\begin{aligned} {k(p(G_s({X}_a^s)),p(G_t({X}_b^t)))}= \sum \limits _{i,j}{k(G_s(x_i^s),G_t(x_j^t)))} \end{aligned}$$

where *a* and *b* denote class labels and $a \ne b$. Each labelled sample in target domain is paired with many labelled samples from different classes in source domain. The distance measure, *d*(, ), is defined as Euclidean distance in the embedded space,

11 $$\begin{aligned} {d(G_s(x_i^s),G_t(x_j^t))}= \frac{1}{2} \left\| G_s(x_i^s)-G_t(x_j^t) \right\| \end{aligned}$$

The similarity measure, *k*(, ), is calculated as

12 $$\begin{aligned} {k(G_s(x_i^s),G_t(x_j^t))}= \frac{1}{2}max\left( 0,m- \left\| G_s(x_i^s)-G_t(x_i^t) \right\| \right) ^2 \end{aligned}$$

Here we use the Frobenius norm and *m* is the margin that specifies the separability in the embedding space. The combination of $L_{\rm{SA}}(G)$ and $L_{\rm{CS}}(G)$ is also known as contrastive loss as defined in [39]. Note that we use the CCSA loss to jointly optimize $G_t$ and $G_s$.

## Experiment Setup

### Datasets

**Fig. 3:**
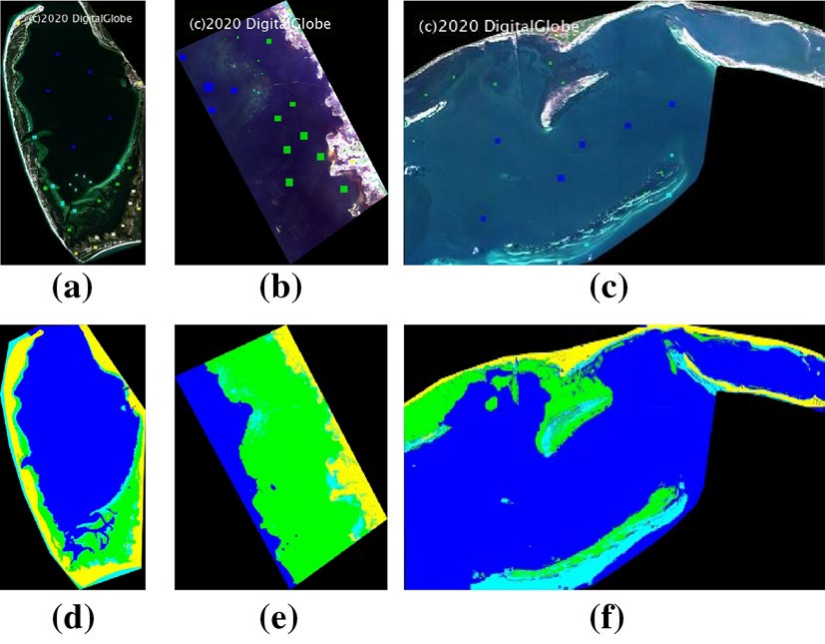
WorldView2 mutlispectral images collected in Florida at **a** SJB **b** KB and **c** SGS. Labelled region colormap: seagrass $\rightarrow$ green, sea $\rightarrow$ blue, sand $\rightarrow$ cyan, land $\rightarrow$ yellow and intertidal $\rightarrow$ magenta. Physics model [41] classification results are shown in **d** SJB **e** KB and **f** SGS

We validated the proposed model on three multispectral images captured by the WorldView-2 satellite at three locations in Florida coastal area: Saint Joseph Bay (SJB), Keeton Beach (KB) and Saint George Sound (SGS). Each image has eight bands (Coastal Blue, Blue, Green, Yellow, Red, Red Edge, NIR-1 and NIR-2) with spatial resolution of 2 meters. An experienced domain expert (co-author of this paper) labelled some regions for five classes in each image: seagrass, sea, sand, land, and inter tidal as shown as green, blue, cyan, yellow and magenta in Fig. 3. Figure 3d–f shows classification results by a physics model [41]. In this study, we trained a deep CNN model at one location and utilize the proposed domain adaptation model to generalize the model to other locations for seagrass detection.

### WorldView-2 Atmospheric Correction

We performed atmospheric correction in the three WorldView-2 satellite multispectral images by matching the images with in situ measurements collected at 22 stations across the images on the same day by a survey boat. At each station, the following measurements were obtained by two spectroradiometer systems in tandem:

- $[E_s(0^+)]$: downwelling spectral irradiance above the sea surface (395 to 795 nm, 2.5 nm bandwidth),
- $L_{\mu }(0.65, \lambda )$: upwelling spectral radiance 0.65 m beneath the sea surface where $\lambda$ represents wavelength [HTSRB, Satlantic Instr.],
- $E_{\mu }(0.21)$ and $L{\mu }(0.21)$: upwelling irradiance and radiance 0.21 m beneath the sea surface [HyperPro, Satlantic Instr.].

With these measurements, we calculated the following attributes:

- Spectral upwelling diffuse attenuation coefficient,
  13 $$\begin{aligned} K_{L_\mu }=-\frac{1}{z}\ln \frac{L_{\mu }(0.65)}{L_{\mu }(0.21)} \end{aligned}$$
  where *z* was the difference in depth between the sensors placed at 0.65 m and 0.21 m.
- Upwelling radiance just beneath the air–water interface $L_{\mu }(0-, \lambda )$ was calculated using $KL_\mu (\lambda )$ to propagate $L_\mu (0.21, \lambda )$ to the surface using Beers Law [42].
- Remote sensing reflectance $[R_{rs}(\lambda )]$ was computed as $L_w (0+, l)/E_s(0+, \lambda )$.

We then reduced the spectral resolution of the field measurements to match the spectral bands of the WorldView-2 image based on the published spectral response functions (www.digitalglobe.com). Finally, we performed a linear regression between the 22 *in situ* measurements to their corresponding WorldView-2 spectra at the same location and created the gain and offset for each band to effectively remove atmospheric signals from the image.

### Data Analysis

We compared the spectral signatures of each class in the multispectral WorldView-2 images taken at different locations. To better visualize the high-dimensional spectral information, we utilized the *t*-distributed stochastic neighbor embedding (*t*-SNE) algorithm [43] to compress high-dimensional data to 2 dimensions.

### *k*-Fold Cross-validation (CV) for Seagrass Detection

At each of the three locations, we performed cross-validation for seagrass detection in the labeled regions. The experimental results gave us performance upper limits for domain adaptation. In *k*-fold CV, we split data into *k* parts and kept one part for testing and the remaining parts for training. We repeated this experiment *k* times such that each part was tested once.

### Domain Adaptation Between Different Locations

In the domain adaptation experiments, each image was used as source image to train a deep CNN model and it was then adapted to other two locations guided by a few labeled samples from the new locations.

### Models for Comparison

#### Source-Only

The source-only model used source domain samples to train a deep CNN model and the model was then directly applied to new locations for seagrass detection.

#### ADDA

Adversarial discriminative domain adaptation (ADDA) [38] adapts the embedding function in the source domain to the target domain based on the GAN loss (Sect. 3.4) with all unlabeled samples in new locations, which was then combined with the classifier trained in source domain to detect seagrass at the new locations.

#### Source + Target

We trained a deep CNN model in the source domain and used a few labeled data samples from the target domain to fine-tune the model. This is a baseline model for transfer learning.

#### CCSA

This model used the contrastive semantic alignment loss and classification loss to learn the embedding function and classification layers [39]. We used two separate embedding functions that were jointly optimized for source and target domains (Sect. 3.5).

#### Proposed Model

We first used the GAN loss to adapt the embedding function trained in the source domain. Then the CCSA loss together with a few labeled samples from target domain was utilized to further adapt the model to new locations as detailed in Sect. 3.5.

## Results

### Data Analysis

**Fig. 4:**
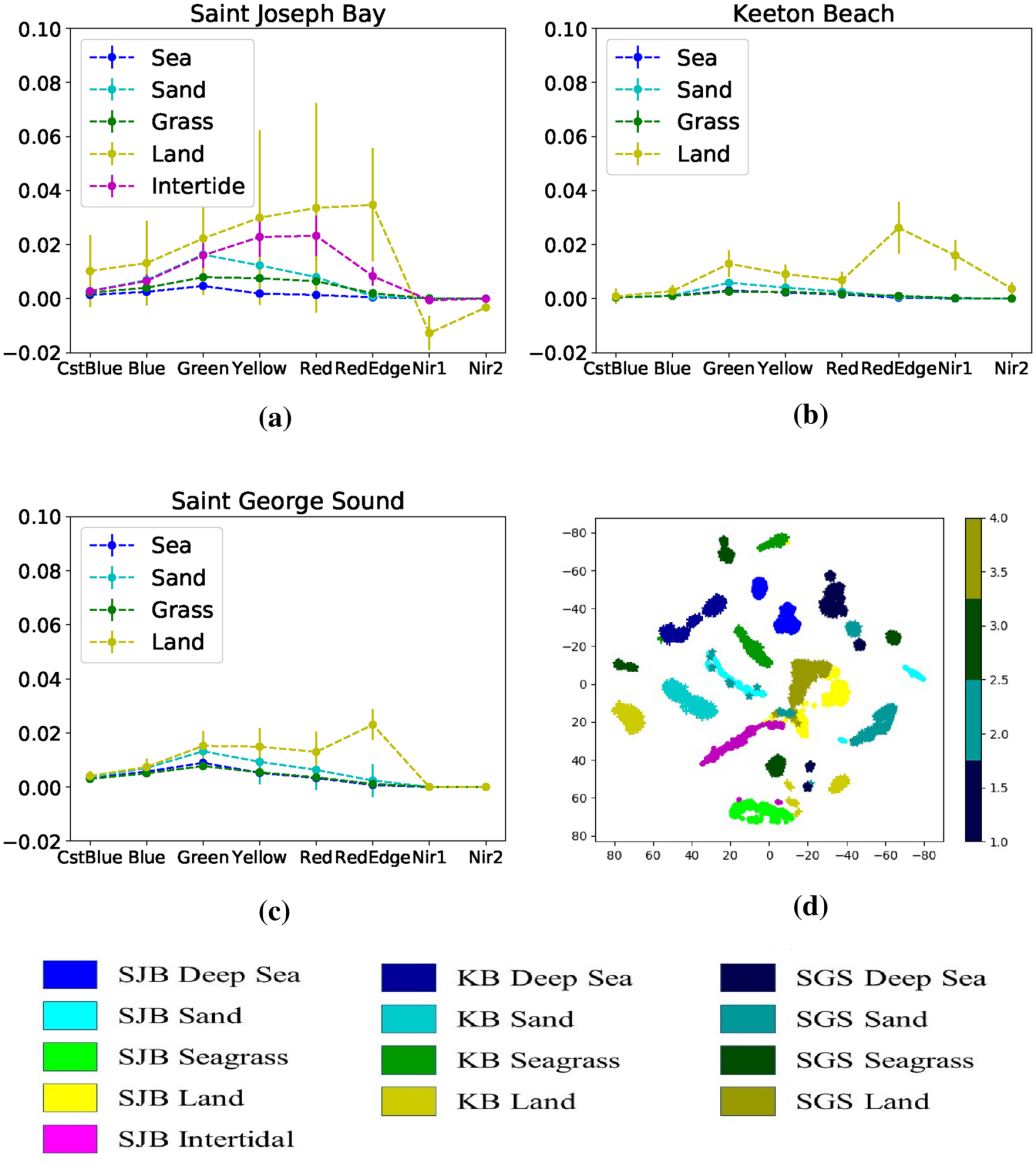
Atmospherically corrected spectral signature, means and standard deviations in mutlispectral WorldView-2 images for different classes shown at **a** SJB **b** KB and **c** SGS. X-axis represents different bands, and Y-axis represents spectral intensity mean and standard deviation. **d***t*-SNE plotting of all three locations for different classes. Green, blue, cyan, yellow and magenta are used to represent seagrass, sea, sand, land and intertidal classes. For the *t*-SNE plotting, the bright shade, dark shade and shade between these two are used to represent SJB, SGS and KB samples, respectively. For seagrass class, we used three different green shades to represent three different locations, e.g.: green, dark green and bright green. Similarly, three shades of yellow, blue, cyan to represent land, sea, and sand classes

Figure 4 shows atmospherically corrected means and standard deviations of the eight WorldView-2 multispectral bands in the labelled regions at the three locations. Land had the highest mean spectral magnitude as it is located above water. Intertidal class is located between sand and land on spectral magnitude. These classes were followed by sand, seagrass and sea in spectral magnitude.

It is also observed that spectral signatures of the same class at different locations have different shapes, indicating that there are distribution shifts among locations. In Fig. 4d, we show *t*-SNE representations for samples from all the three locations. We use green, blue, cyan, yellow and magenta to represent seagrass, sea, sand, land and intertidal classes. We use three shades to represent three different locations: the most bright shade, most dark shade and shade between this two to represent SJB, SGS and KB samples, respectively (Fig. 4d). Note that there are significant distribution shifts among different locations in different classes.

### Hyper-Parameter Determination

Deep CNN models take a patch from the multispectral image to predict a class label for the central pixel of the patch. A large patch may cause over-smoothing and requires higher computation power, whereas a too small patch may degrade the performance. After some trial and error, we found that a $5\times 5\times 8$ patch size produced the best results in the threefold CV experiment. Other parameters were determined in the same way and are listed below.

#### Embedding Functions $G_s$ and $G_t$, in CNN Models

Both contain two convolutional layers followed by a flatten layer. The first layer had 20 filters with a size of 2 * 2 * 8, and the second layer had 100 filters with a size of 4 * 4 * 20. All layers used ReLu activation function.

#### Classifiers $C_s$ and $C_t$, in CNN Models

Both contained a fully connected layer with 84 hidden units, and the output layer had 5 units with SoftMax activation function for classification.

#### Source and Target Data Pairing

400 labeled samples from each class in source domain were randomly selected to pair with the few labeled samples in target domain to compute the loss function described in Sect. 3.5.

#### Training Parameter Settings

We trained the source CNN models 50 epochs with a batch size of 128. We trained the unsupervised adversarial domain adaptation step 300 epochs and the CCSA step 240 epochs in all experimentals.

#### Learning Rate

We used 0.0002 as the learning rate in all experiments. No dropout layer was used.

### Cross-validation

Table 1 shows threefold CV results at the three locations to find upper limits of domain adaption. We achieved 99.99% accuracy at SJB, 99.98% at KB and 99.71% at SGS, respectively. The low variances indicate that the results are very reliable.

**Table 1:** Threefold cross-validation results at SJB, KB and SGS

Fold no.	SJB (%)	KB (%)	SGS (%)
1st Fold	99.99	99.98	99.83
2nd Fold	99.99	99.98	99.66
3rd Fold	99.99	99.97	99.64
Mean	99.99 ± 0.00	99.98 ± 0.01	99.71 ± 0.10

### Domain Adaptation

**Table 2:** Classification results in target domain by different methods (All numbers are in %)

Numb of shots	Tasks	SJB $\rightarrow$ KB	KB $\rightarrow$ SJB	SJB $\rightarrow$ SGS	SGS $\rightarrow$ SJB	KB $\rightarrow$ SGS	SGS $\rightarrow$ KB
N/A	Source Only (Baseline)	34.75	45.00	25.08	74.04	15.91	64.14
N/A	ADDA	35.76	42.20	67.80	35.39	78.69	99.43
1-shot	Source+Target (f.t.)	$84.78\pm 18.09$	$76.21\pm 17.64$	**79.98 **± **15.05**	$74.23\pm 16.61$	$63.39\pm16.70$	$71.13\pm 6.85$
	CCSA	$71.26\pm 5.43$	$78.60\pm 6.95$	$73.34\pm 7.09$	$76.70\pm 5.65$	$72.49\pm 1.77$	$70.82\pm 4.44$
	Proposed Model	**98.84** ± **0.29**	**86.12** ± **3.55**	$71.35\pm 17.20$	**80.23** ± **3.04**	**93.32** ± **1.75**	**99.35** ± **0.09**
2-shot	Source+Target ((f.t.)	$84.78\pm 18.09$	$76.21\pm 17.64$	$79.98\pm 15.05$	$74.23\pm16.61$	$63.39\pm16.70$	$71.13\pm6.85$
	CCSA	$82.56\pm 20.30$	$87.47\pm 3.30$	$88.87\pm 7.50$	**90.79** ± **1.68**	$84.84\pm3.65$	$84.31\pm20.89$
	Proposed Model	**99.30** ± **0.14**	**91.72** ± **5.88**	**89.65** ± **6.85**	$89.70\pm5.34$	**91.55** ± **6.98**	**99.45** ± **0.08**
3-shot	Source+Target (f.t.)	$81.88\pm 15.94$	$84.80\pm 11.38$	$90.47\pm8.37$	$76.36\pm21.78$	$72.96\pm2.12$	$67.27\pm7.03$
	CCSA	$83.95\pm 21.08$	$88.83\pm 2.76$	**90.84** ± **8.39**	$87.68\pm5.17$	$89.26\pm6.91$	$87.27\pm21.61$
	Proposed Model	**99.32** ± **0.72**	**94.28** ± **1.90**	$89.46\pm7.13$	**92.22** ± **4.68**	**95.20** ± **1.23**	**99.42** ± **0.07**
4-shot	Source+Target (f.t.)	$87.17\pm19.10$	$85.60\pm11.86$	$67.49\pm31.52$	$71.14\pm18.34$	$75.78\pm18.24$	$65.15\pm0.10$
	CCSA	$96.82\pm3.76$	$95.26\pm4.24$	**90.93** ± **8.31**	$94.79\pm5.24$	$91.19\pm7.72$	$98.67\pm1.05$
	Proposed Model	**99.44** ± **0.46**	**96.31** ± **2.04**	$90.92\pm8.00$	**96.84** ± **1.76**	**92.38** ± **6.83**	**99.38** ± **0.12**
5-shot	Source+Target (f.t.)	**99.88** ± **0.07**	**98.20** ± **1.11**	$67\pm30.39$	$92.40\pm5.45$	$71.07\pm0.40$	$64.58\pm0.66$
	CCSA	$99.72\pm0.30$	$95.48\pm4.22$	**91.01** ± **8.14**	$95.26\pm5.56$	$91.38\pm7.99$	$99.43\pm0.33$
	Proposed Model	$99.07\pm0.33$	$95.50\pm2.84$	**91.01** ± **8.00**	**96.27** ± **1.43**	**93.93** ± **4.67**	**99.47 ± 0.12**
10-shot	Source+Target (f.t.)	**99.57** ± **0.67**	$86.01\pm22.57$	$89.02\pm15.09$	$80.91\pm15.91$	$71.03\pm0.10$	$76.08\pm20.30$
	CCSA	$99.42\pm0.44$	$99.04\pm0.42$	$97.71\pm0.82$	**98.73** ± **0.58**	$97.67\pm1.19$	$99.56\pm0.25$
	Proposed Model	$99.34\pm0.31$	**99.09** ± **0.05**	**98.38** ± **0.87**	$98.69\pm0.93$	**98.33** ± **0.63**	**99.59** ± **0.32**
N/A	3-fold CV	$99.98\pm0.01$	$99.99\pm0.00$	$99.71\pm0.10$	$99.99\pm0.00$	$99.71\pm0.10$	$99.98\pm0.01$

We conducted six domain adaptation experiments for the three WorldView-2 satellite images as KB $\rightarrow$ SJB, SJB $\rightarrow$ KB, SGS $\rightarrow$ SJB, SJB $\rightarrow$ SGS, SGS $\rightarrow$ KB and KB $\rightarrow$ SGS. Comparison of our proposed model with previous models and results is shown in Table 2. For each domain adaptation experiment, we implemented 6 scenarios including 1 to 5-shot and 10-shot cases (*n*-shot stands for having *n* labeled samples from each class). One “shot” means one labeled sample per class in target domain is used to adapt the model. Each scenario was performed three times with randomly selected labelled samples from target domain, and means and standard deviations are shown in Table 2. The proposed method achieved the best results in 28 out of 36 scenarios in Table 2. In the 10-shot domain adaptation scenario, the proposed method approached to model upper limits (3-fold CV performances). The second best model is the *Source+Target* (*f.t.*) that achieved the best results in 4 out of 36 scenarios in Table 2.

### *t*-SNE Plotting

**Fig. 5:**
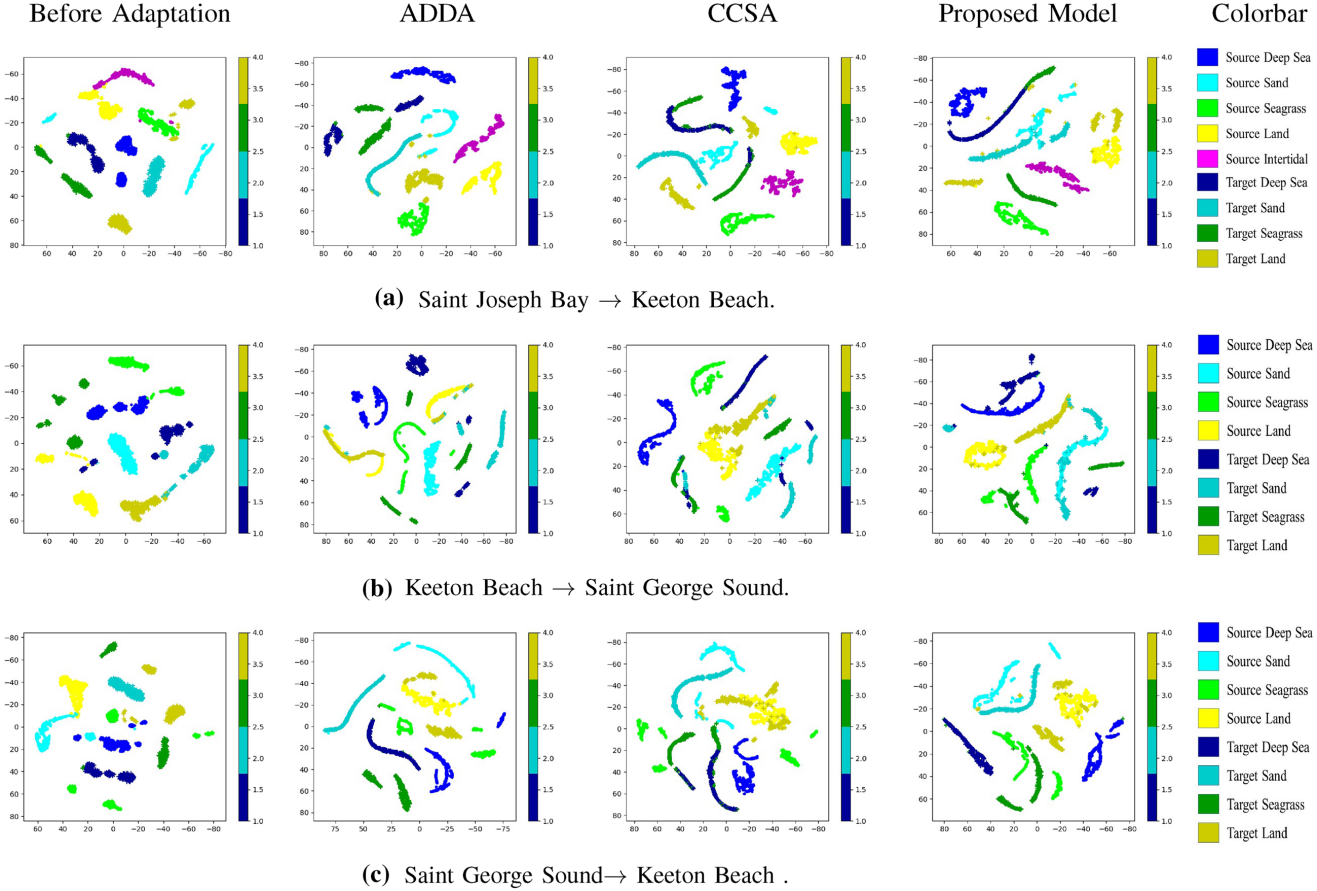
*t*-SNE plots in embedding space after 1-shot domain adaptation in target domain. **a** SJB $\rightarrow$ KB **b** KB $\rightarrow$ SGS and **c** SGS $\rightarrow$ KB. Green, blue, cyan, yellow and magenta are used to represent seagrass, sea, sand, land and intertidal, respectively. The most bright shade and the most dark shade are used to represent source and target domain samples, respectively

We demonstrate how the proposed model maps samples from different domains to the embedding space by utilizing the *t*-SNE algorithm with the following procedure:

1. Compress the original samples from source and target domains (200 = 5*5*8 dimensions) to 2 dimensions using the *t*-SNE algorithm (before adaptation),
2. Feed original samples from source and target domains to the embedding functions, $G_s$ and $G_t$, respectively, to obtain new representations in the embedding space,
3. Compress the new representations to 2 dimensions using the *t*-SNE algorithm (after adaptation),
4. Plot the compressed data samples on 2D plane using different colors for different classes. Use blue, cyan, green, yellow and magenta colors to represent sea, sand, seagrass, land and intertidal class. Utilize two different shades of same color to denote target and source samples

*t*-SNE results are shown in Fig. 5 for three domain adaptation scenarios: SJB $\rightarrow$ KB, KB$\rightarrow$ SGS and SGS $\rightarrow$ KB. We used 400 samples in each class, respectively, from source and target domains. The proposed model achieved better embedding for sea and seagrass classes as compared to CCSA model in the scenario of SJB $\rightarrow$ KB as shown in Fig. 5a. In Fig.  5b, c, similar trends are observed for KB $\rightarrow$ SGS and SGS $\rightarrow$ KB cases. The CCSA model incorrectly mapped seagrass samples closer to sea samples and sand samples in the embedding space. Unsupervised domain adaptation method was performed poorly in all the cases as shown in Fig. 5.

### Ablation Study

**Table 3:** Ablation study of the proposed method

Shots	Methods	KB $\rightarrow$ SGS (%)
	ADDA	78.69
1-shot	CCSA	$72.49\pm 1.77$
	Proposed Model w/o Joint Optimization	$84.77\pm 6.61$
	Proposed Model	**93.32** ± **1.75**
2-shot	CCSA	$84.84\pm 3.65$
	Proposed Model w/o Joint Optimization	$91.23\pm 8.87$
	Proposed Model	**91.55** ± **6.98**
3-shot	CCSA	$89.26\pm 6.91$
	Proposed Model w/o Joint Optimization	$91.41\pm 7.89$
	Proposed Model	**95.20** ± **1.23**
4-shot	CCSA	$91.19\pm 7.72$
	Proposed Model w/o Joint Optimization	$90.58 \pm 5.21$
	Proposed Model	**92.38** ± **6.83**
5-shot	CCSA	$91.38\pm 7.99$
	Proposed Model w/o Joint Optimization	$82.17\pm 16.75$
	Proposed Model	**93.93** ± **4.67**

Our proposed model contained two loss functions: semantic contrastive alignment loss and GAN loss. If we remove the GAN loss from the proposed model and just use semantic contrastive alignment loss for domain adaptation, the model would be equivalent to the CCSA model. If we remove the contrastive semantic alignment loss from the proposed model, then it will be equivalent to the unsupervised ADDA model. Our proposed model also used joint optimization for the source embedding function, $G_s$, and the target embedding function, $G_t$, in the supervised domain adaption step. We investigated the three components in the ablation study for KB $\rightarrow$ SGS and results are shown in Table 3. Note that ADDA does not require labelled samples from target domain, so only one scenario was performed. The proposed model with all the three components achieved the best results.

### Classification Maps

**Fig. 6:**
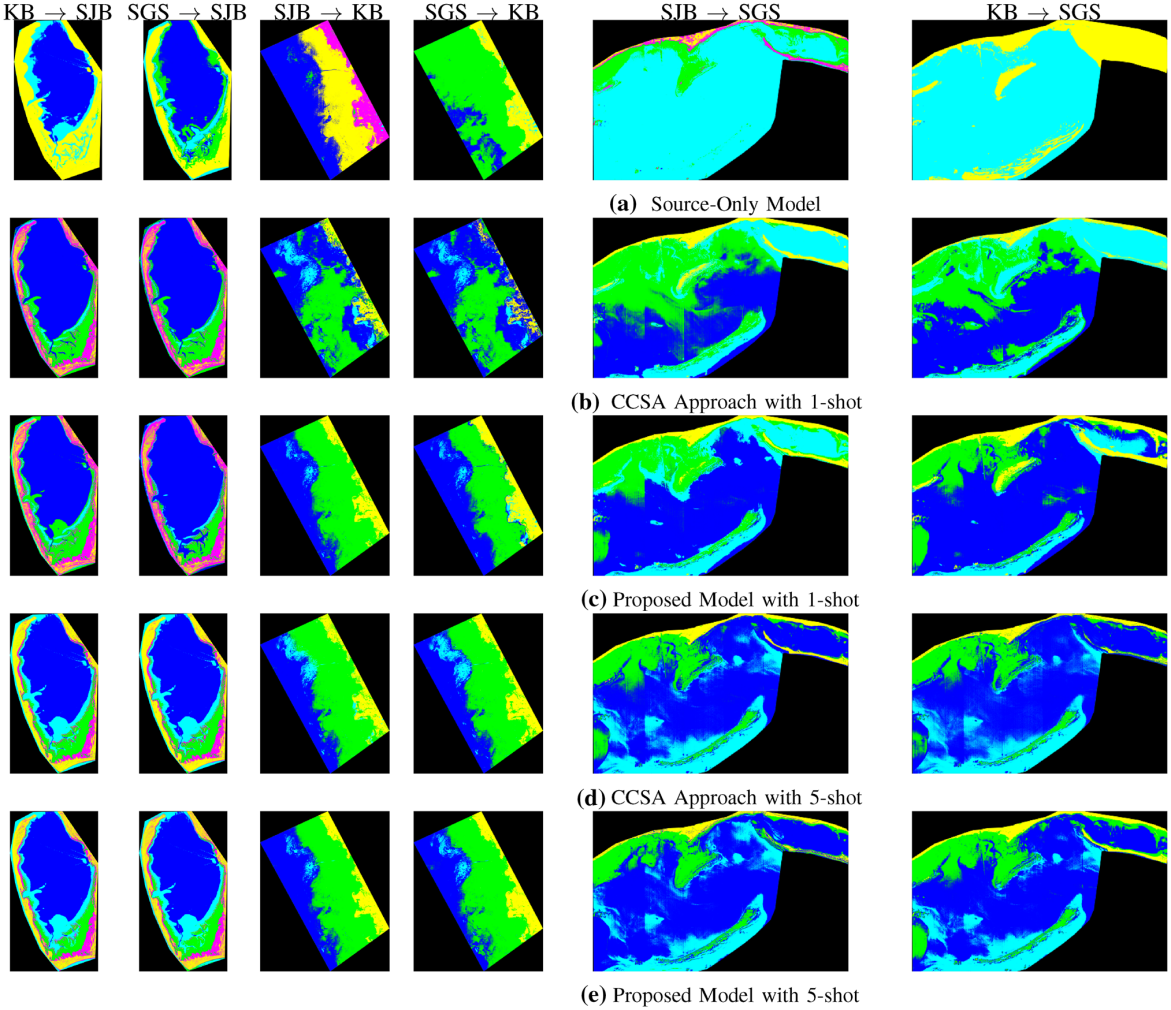
End-to-end classification maps produced by domain adaptation based on **a** source model **b** CCSA model with 1-shot **c** proposed model with 1-shot **d** CCSA model with 5-shot and **e** proposed model with 5-shot

The classification maps produced by our proposed model, CCSA approach, and baseline model are shown in Fig. 6. The first row of Fig. 6 represents the base line classification maps where we directly applied classification models trained in source domains to classify target domain images without performing any adaptation. The baseline model performed poorly as compared to the physics model as shown in Fig. 3. Second and fourth rows of Fig. 6 represent classification maps produced by CCSA model with 1-shot (Fig. 6b) and 5-shot (Fig. 6d), respectively. In this step, we used only contrastive semantic alignment loss to perform domain adaptation task. The third and the last row in Fig. 6 represents classification results by the proposed model with 5-shot. We used both the GAN loss and the contrastive semantic alignment loss for domain adaptation. The proposed model with 5-shot produced good classification results as compared to the physics model as shown in Fig. 3. Note that the classification maps shown here are for visualization purpose only as the physics model has 10% error [41].

## Discussion

Our proposed approach produced the best results for 28 out of 36 domain adaptation experimental scenarios as shown in Table 2. For KB $\rightarrow$ SGS and SGS $\rightarrow$ KB, our proposed method won all the scenarios. For SJB $\rightarrow$ KB, our model achieved 98.84% accuracy using just one labelled sample from target domain and it is much better than CCSA (71.26%), ADDA (35.76%) and *Source+Target* (*f.t.*) (84.78%). For KB $\rightarrow$ SGS, our proposed model with one labelled sample from target domain achieved an accuracy of 93.32% as compared to CCSA (72.49%), ADDA (78.69%) and *Source+Target* (*f.t.*) (63.39%). Similar trends can also be found in SGS $\rightarrow$ KB in all the 1-shot domain adaptation cases except SJB $\rightarrow$ SGS, where all the methods achieved similar results. On average, our proposed method won by a large margin.

As we utilizing more labeled samples from the target domain, the proposed method can still provide better domain adaptation, winning four or five out of the six experimental scenarios with 2-shot up to 10-shot cases. On average, however, the winning margin decreased as more labeled samples were used for adaptation. For the 10-shot scenario, CCSA and the proposed method achieved similar results and the results were close to the threefold CV results, indicating that adding more labeled samples from target domain did not provide more benefits.

For most of the scenarios, standard deviations of the proposed method were much smaller than these of other methods. Our method first utilized a large number of unlabeled samples in both domains to perform domain adaptation. We then used a few labeled samples from target domain to semantically align class specific distribution in the embedding space. The first step of the method aligned marginal distribution based upon a large number of unlabeled data and worked as a regularizer for the subsequent semantic alignment. Therefore, the proposed method can provide more stable performances.

Figure 5 shows *t*-SNE plots for data samples or embeddings in source and target domains before and after domain adaptation. Before adaptation, we can see that data distributions in source domain and target domain are not aligned. ADDA aligned distributions between the source and target domains, but there is no guarantee that the same class samples from different domains will be mapped closer in the embedding space. With the guidance of labelled samples, CCSA and the proposed model can do a better semantic alignment: same class samples from different domains can be mapped closer, and the proposed method can do a better job as compared to CCSA.

We only performed the KB $\rightarrow$ SGS case study for ablation as shown in Table 3. All three components in the proposed model are important. With joint optimization, the proposed model became much more stable and achieved much smaller standard deviation in performances for all the scenarios. With more labeled samples from target domain, CCSA can perform much better than ADDA.

As compared to the physics model classification maps in Fig. 3d–f, the classification maps produced by the proposed model with 5-shots were much better than those from the direct source domain model as shown in Fig. 6a. Classification maps produced by CCSA with 5-shot (Fig. 6d) are good. However, those produced by CCSA with 1-shot (Fig. 6b) are much worse. Note that the physics model results have 10% error [41] and the classification maps are shown for visualization purpose only. For accurate quantitative assessment of these models, please see results in Table 2 where the accuracy was computed in the labeled regions.

## Conclusion

Automatic seagrass detection systems in multispectral images are important tools for seagrass monitoring. Labelling atmospherically corrected multispectral images is labor intensive and time consuming. We developed a semi-supervised domain adaptation method for deep CNN models for seagrass detection. The proposed model first used unlabelled samples in both domains to adapt source domain model to target domain based on the GAN loss. Then it utilized contrastive semantic loss with a few labelled samples from target domain to further adapt the model. In addition, the source model and target model were jointly optimized in the second step. We evaluated the proposed model in three atmospherically corrected WorldView-2 multispectral images taken in Florida and achieved the best results among 28 out of 36 experimental scenarios. Future work will evaluate the proposed model with other image detection methods across broader regional areas such as the southeastern USA.
